# Young Love “Locked Down”: Adolescent and Young Adult Perspectives on Sexting During the Covid-19 Pandemic in England

**DOI:** 10.1007/s10508-023-02734-z

**Published:** 2023-11-15

**Authors:** Emily Setty, Emma Dobson

**Affiliations:** 1 Department of Sociology, 11 AD 03, University of Surrey, Guildford, Surrey, GU2 7XH UK; 2 School of Education, University of Durham, Durham, UK

**Keywords:** Adolescents, Young adults, Sexting, Lockdown, Consent, Covid-19

## Abstract

There were limited opportunities for in-person social, intimate, and sexual interactions in England during 2020–2021, due to restrictions imposed by the UK government in response to the Covid-19 pandemic. While previous studies examined the effects of lockdown on intimate relationships, there is less qualitative research regarding young people’s perspectives on and experiences of digitally mediated intimacy (sexting) during the period. This paper discusses findings from focus groups with 80 adolescents and interviews with 38 young adults that explored the topic. Analysis identified a normalization of non-consensual distribution of intimate images within adolescent peer culture and a reluctance to report or intervene in response to incidents of non-consensual distribution that are witnessed or experienced. The adolescent girls and young adult women also described other forms of unwanted and invasive image-sharing and requests for images. Young adults held various perspectives on sexting during lockdown, with some describing sexting as unfulfilling and/or “risky” and others sharing experiences of using sexting to generate intimacy and, among some, engaging in unwanted sexting with partners. By considering both adolescent and young adult perspectives obtained through focus groups and interviews, the study highlighted how group-level norms and meanings surrounding the risks and rewards of sexting may be reproduced or reworked as individuals transition from adolescence to young adulthood. The study underscores the need to support adolescents and young adults in cultivating healthy digital sexual cultures and interpersonal relationships.

## Introduction

Opportunities to interact socially, intimately, and sexually in-person were limited in England during 2020–2021, due to restrictions imposed by the UK government in response to the Covid-19 pandemic. Restrictions included limitations on the legal right to leave one’s home, to associate in public space with people outside the home, and the requirement to socially distance (i.e., to maintain a distance of at least two meters from others outside the home). Consequently, there was a de facto criminalization of sexual activity between individuals who did not live together. While involvement in sexual activity differs between and through adolescence and young adulthood (Meier & Allen, 2009), these age groups are least likely to co-habit and, therefore, would have been significantly affected by the regulations (Wignall et al., 2021). Many young people—notably adolescents but also some young adults—were, moreover, home-confined within families and thus experienced reduced independence and autonomy, including in relationships (Hall & Zygmunt, 2021).

Romantic and intimate relationships are important to young people’s socioemotional development during both adolescence and young adulthood (Collins et al., 2009). Relationships are robust predictors of health and well-being (Pietromonaco & Beck, 2019), including during times of stress (Pietromonaco & Collins, 2017). While lockdown is likely to have disrupted this formative period (Lindberg et al., 2020), many activities moved online, including the maintenance of sexual and romantic relationships (Lindberg et al., 2020). This paper discusses young people’s perspectives on and experiences of hosting sexually intimate interactions online during lockdown, based on one-to-one interviews with young adults and focus groups with adolescents conducted in England during 2021–22.

We take a critical realist approach to identifying how lockdown (re)created and (re)shaped conditions for sexting among young people (Scott et al., 2020). The findings provide insight into patterns of consensual and non-consensual sexting and indicate that group-level norms and meanings about adolescent sexting culture, as expressed by adolescents in focus groups, may be variously reproduced and reworked by young adults as they narrate (inter)personal perspectives and experiences in interviews. We suggest the findings reflect both the age categories (adolescence vs. young adulthood) and the methods used (focus groups vs. interviews) to generate the data.

### Digital Intimacies and Sexting

Young people’s sociosexual lives and development have become increasingly digitally mediated with the advent of internet-enabled devices that present opportunities for sexually intimate interactions online with people they do and do not know offline. The term “digital intimacies” encompasses the ways young people forge connections, build intimacy, and manage interpersonal relationships online (Scott et al., 2020). These include sharing sexually explicit content through (asynchronous) messages, images, videos and (synchronous) video/voice calls, colloquially termed “sexting.” Studies suggest sexting increases during adolescence, reaching a peak in young adulthood (Mori et al., 2020).

Nuanced perspectives on sexting (Drouin et al., 2017; Lee & Crofts, 2015) suggest that it presents opportunities and risks (Lunde & Joleby, 2021), is not always harmful, may take place consensually, and, for adolescents, may sometimes be “developmentally normative” (Wolak & Finkelhor, 2011). Motivations for sexting vary and relate to self-expression, experimentation, and intimate connection which sometimes relate to but go beyond sexual arousal and fulfillment (Anastassiou, 2017; Cooper et al., 2016). Sexters are aware of risk and consider potential dangers and benefits (Howard et al., 2022), with it being experienced as both exciting and risky (Hunehäll Berndtsson & Odenbring, 2021). While sexting may not replace in-person interaction, it can help “romantic partners stay connected when they cannot see each other in-person” (Johnstonbaugh, 2022, p. 67), with implications for sexting during the pandemic.

#### Consensual and Non-Consensual Sexting

Experiences and outcomes of sexting rest upon consent and privacy (Doyle et al., 2021). Adolescents have been found to organize their perspectives on the positive and negative aspects of sexting around consent (Meehan, 2022) while young adults perceive sexting as “intimate” and, therefore, as deserving of privacy (Hasinoff & Shepherd, 2014). Among young adults, Johnstonbaugh (2022) found that when sender and recipient are “on the same page” (p. 62), sexting can be mutually pleasurable and satisfying. This was not the case, however, if unwanted by one person within the interaction or when involving non-consensual practices. Non-consensual sexting affecting both adolescents and young adults includes unwanted solicitation for images and pressure to produce images, sending unwanted images (“cyber-flashing” or “dick pics”), non-consensual further distribution of images, and faked or non-consensually recorded or produced images (e.g., “up-skirting” or “down-blousing”) (Ringrose et al., 2021; Setty et al., 2022).

Evidence suggests that patterns of consensual and non-consensual image sharing are gendered; adolescent girls and young adult women are disproportionately likely to report being victimized and boys and young men are, in turn, disproportionately likely to perpetrate non-consensual and abusive sexting (Foody et al., 2021; Henry et al., 2018; Ringrose et al., 2021; Wolak et al., 2018). Girls and young women are, in turn, more likely to describe feeling uncomfortable and as having fewer positive experiences and more trauma after sexting than boys and young men (Drouin et al., 2017; Samimi & Alderson, 2014).

Girls and young women experience relatively more pressure to sext, with any concerns about negative consequences, such as non-consensual further distribution, becoming secondary to decision-making because of a reluctance to displease their partners (Howard et al., 2022; Thomas, 2018). They are then at relatively greater risk of having their images distributed further without their consent by boys and young men who stand to gain social capital from collecting and sharing the images in their male peer groups (Foody et al., 2021; Kernsmith et al., 2018; Ringrose et al., 2012, 2021; Setty, 2019). Among adolescents, gender double standards mean girls experience stigma and shame if exposed for sexting (Ringrose et al., 2012) and judgments from peers, even family, acts as a form of social control over girls (Pavón-Benítez et al., 2022). Girls have, in turn, been found to describe safety behaviors when sexting such as keeping identifying features out of images (Meehan, 2022), whereby safer sexting is about stigma management (Ouytsel et al., 2021).

Both girls and young women also experience being sent unwanted and unsolicited images from boys and men. While Hayes and Dragiewicz (2018) conceptualize it as a form of gendered sexual aggression, girls and young women often trivialize it as merely “annoying” or “irritating” rather than as a form of abuse (Bonilla et al., 2021; Setty et al., 2022; Thorburn et al., 2021). These studies find that refusing to reciprocate is often met with further abuse and girls and women typically manage the risk by blocking the sender.

#### The Gray Areas of Consent, Wanting, and Willingness

Reports of consensual image sharing are more common than aggravated or abusive sharing, particularly among young adults (Bianchi et al., 2016, 2017). Yet, studies identify a continuum of consent, spanning both direct and indirect pressure and coercion (e.g., Cooper et al., 2016; García-Gómez, 2017; Ringrose et al., 2021; Setty, 2019; Thomas, 2018; Thorburn et al., 2021). Laird et al. (2021) distinguish sexting in terms of Morgan et al.’s (2006) model of forced, coerced, and willing sex. They locate willing but unwanted sexting in terms of social and relational expectations, obligations, and pressures, with young adult women reporting more coerced and willing but unwanted sexting than did the men. They found that a diminished self of self in relationships and over-identification with others mediated the association between gender and sexting, suggesting that non-consensual sexting may involve women prioritizing the needs and requests of men.

Amundsen (2022) describes sexting as entailing “emotion work” or “mediated intimacy work” for women. Prioritizing male sexual pleasure and desire is normalized in terms of men’s perceived needs for sex (Bonilla et al., 2021) while also resulting in less satisfaction and more discomfort for women who feel obligated to engage in sexting regardless of personal desire (Johnstonbaugh, 2022). Among adolescents in Northern Ireland, Agnew (2021) identified how the symbolic power of sexting to demonstrate love, trust, and commitment may be coercive for girls who are pressured to share images to affirm their feelings for their male partner. Girls and young women typically resist victimization discourses, however, and sometimes claim empowerment, including when describing unwanted but chosen sexting, notwithstanding the further privileging of boys’ and men’s needs and the ‘male gaze’ it represents (Bonilla et al., 2021; Setty, 2020).

While gendered dynamics of abusive sexting suggest it may constitute a form of gender-based violence (Henry & Powell, 2018; McGlynn et al., 2017; Thorburn et al., 2021), it is important to avoid reifying or essentializing about girls and women and boys and men (see Elliott, 2014). The belief that boys and men are inherently sexually driven and girls and women inherently vulnerable has led to holding the latter responsible for managing boys’ and men’s sexual desires and blaming them in the event of abusive sexting because male perpetration is normalized and, therefore, deemed up to potential victims to predict and remedy. There also ensues a corresponding expectation that boys and men will be interested in sexting and, therefore, an association between sexting and masculinity which may create pressure for males and prevent recognition of abuse or victimization perpetrated against them (Agnew, 2021; Hunehäll Berndtsson & Odenbring, 2021; Ravn et al., 2021; Ringrose et al., 2012; Setty, 2020).

#### Challenging and Reporting Non-Consensual Sexting

Studies identify fatalism about abusive sexting and limited reporting and bystander intervention particularly among adolescents (e.g., Grobbelaar & Guggisberg, 2018; Lloyd, 2019; Ouytsel et al., 2021; Ravn et al., 2021; Setty, 2019, 2020). Harder (2021) found that adolescents experience pressure not to intervene in order to display social and cultural competency and, therefore, to maintain inclusion among peers. Victim blaming is common, because of the belief that abuse is best prevented by not sharing to begin with and, therefore, that victims are responsible. Reluctance among adolescents to report abusive sexting to adults relates to fears of punishment (Jørgensen et al., 2019; Phippen, 2017; Setty, 2020) and a perceived lack of safe and non-judgmental avenues to report abuse (Dodge & Lockhart, 2022; Jørgensen et al., 2019; Ringrose et al., 2021).

Poor awareness about abuse and reluctance to report has been linked to risk averse educational interventions delivered to adolescents (e.g., in Relationships and Sex Education in schools) that emphasize the illegality of sexting for minors (Phippen, 2017; Ringrose et al., 2021) and utilize a ‘pedagogy of regret’ to deter sexting (Albury, 2014). These approaches conflate consensual and non-consensual sexting and position the latter as a consequence of the former rather than as abuse (Krieger, 2017; McGlynn & Rackley, 2017). Alternative conceptualizations of rights-based sexting education instead focus on privacy and consent, challenge shame and blame of victims (Crofts & Lieven, 2017), and support young people to develop ethical digital sexual cultures that can be carried into adulthood (Setty, 2020).

#### Sexting During Lockdown

Surveys suggest that in-person intimacy and sexual activity with partners fell during the pandemic (e.g., Lehmiller et al., 2021; Lindberg et al., 2020; Wignall et al., 2021) although did not completely stop (e.g., Coombe et al., 2021; Sanchez et al., 2020). Adolescents and younger adults reported relatively more declines in sexual activities and less sexual satisfaction than before lockdown (Mercer et al., 2022). Reduced sexual intercourse and bonding was associated with loneliness and depressive symptoms among some individuals, while those living with parents were particularly affected because of parental monitoring and reduced independence and privacy (Stavridou et al., 2021). For young adult couples living apart, social distancing measures increased feelings of loneliness, due to decreased opportunities for physical and social intimacy (Lehmiller et al., 2021). Those dating or in a relationship spent less time with partners in-person and some experienced relationship conflict due to social distancing (Yarger et al., 2021).

Public health institutions recommended online sexual activities as a safer alternative to in-person interactions during the pandemic (see Alpalhão & Filipe, 2020). Some surveys suggest online sexual behaviors rose during lockdown (e.g., Ballester-Arnal et al., 2021; Lehmiller et al., 2021; Vendemia & Coduto, 2022). There was more sexual image sharing (e.g., Thomas et al., 2022), consumption of pornography (e.g., Mestre-Bach et al., 2020) and online dating (Sanchez et al., 2020). Sexting during lockdown was associated with more satisfaction with one’s sex life among young adults, and rates were higher among those experiencing more pandemic-related stress, those not living with a partner (Lehmiller et al., 2021), and those living away from their family home (Romero-Rodríguez et al., 2022).

Thomas et al. (2022) reported an association between social isolation and willingness to participate in sexting during lockdown among young adults, independent of “trait loneliness,” suggesting it was a situational rather than dispositional association. Both single and coupled respondents used sexting to cope with isolation, including those concerned about privacy, suggesting a salience of social needs over privacy concerns. Sexting acted as a coping mechanism for some young adults (Luo & Hancock, 2020), albeit a potentially adaptive or maladaptive one depending on context (Bianchi et al., 2021). Some surveys, however, found little (Romero-Rodríguez et al., 2022) or no difference in sexting rates pre and during pandemic (Yarger et al., 2021) or a reduction in rates during, compared to before, the pandemic (Gassó et al., 2021).

### Current Study

While there are quantitative data available on young adults’ experiences of relationships and digital intimacies during lockdown, there is less evidence regarding adolescent sexting. Data about children’s online behavior in England and Wales (ONS, 2021) found that 10% of 13–15-year-olds reported having received a sexual message and girls were significantly more likely to have done so than boys. Moreover, while surveys identify trends and patterns among young adults, they do not provide insight into meanings and experiences. This paper addresses these gaps by discussing qualitative data regarding adolescents’ and young adults’ perspectives on sexting during lockdown, including regarding how dynamics of consent and privacy unfolded at a time when in-person interaction was limited.

We examine participants’ perceptions, attitudes, and experiences regarding consensual and non-consensual acts of sexting as unfolded during, while also transcending, the lockdown period as a condition of their lives. We base this examination on data generated from focus groups and interviews that allowed, respectively, adolescents to articulate the social norms and meanings surrounding sexting and adults to share personal perspectives on and experiences of sexting. Typically, studies of adolescent sexting, for ethical reasons, examine participants’ perceptions of sexting and so pertain more to social beliefs rather than personal motivations or practices (Crofts et al., 2015). Furthermore, experiences of sexting likely differ between younger adolescents, older adolescents, and young adults (Dully et al., 2023). Hence, we include both adolescents’ and young adults’ perspectives and consider the conditions in which sexting is given meaning and experienced, including in adolescent sexting culture and the intra- and interpersonal contexts of intimacy, expectation, and obligation in youth adult relationships.

## Method

### Participants

To explore how relationships were affected by lockdown and the role of technology in creating and sustaining intimacy in relationships during the period, we interviewed 38 young adults (aged 18–24) and held 14 focus groups with 80 adolescents (aged 13–20) during late 2021–May 2022 (total = 118 participants). A qualitative approach supported the research aim to go beyond quantifying the extent and forms of sexting among young people during lockdown to instead exploring motivations, feelings, and experiences.

### Procedure and Measures

Originally, both adolescents and young adults were invited to participate in the method of their choice (interview or focus group). Each method was intended to offer different but valuable insights into participants’ perspectives and experiences (personal experiences and stories vs. group-level social meanings and norms) and we wanted participants to select whichever method they felt most comfortable with. Ultimately, however, young adults all opted to take part in interviews and adolescents all opted for focus groups. This may be because the adolescents felt more confident and willing to be a part of a group (and they had the option to form groups of friends), while most of the young adults put themselves forward as individuals and were content to share their story in an interview setting. We draw on data from both methods, while acknowledging and exploring the nature of the insights obtained through the different methodological stances and how they may reflect the methods and the age ranges of participants.

Young adults were recruited via social media and university communication channels. Participants responded to the advert and were provided with an information sheet. Those who wanted to participate completed and returned a consent form and a date/time for the interview was scheduled. For the young adult sample (*n* = 38), more females (*n* = 26/38) than males participated, although the sample was diverse regarding ethnicity (BAME: *n* = 21/38) and somewhat regarding sexual orientation (LGB+ *n* = 10/38).

Adolescents were recruited through schoolteachers and an LGBT+ youth club leader. They were provided with information sheets and those who wished to participate signed consent form, with parental consent also required for those under 16, except in the LGBT+ youth club group where the youth club leader attested to the competence of the young people to consent to participate without parental consent because for most, their involvement with the youth club was not known to their parents and it may have been unsafe to seek parental consent.

The adolescents were mostly white (*n* = 67/80) and heterosexual (*n* = 46/80). There was a mix of males (*n* = 38/80) and females (*n* = 36/80), with six non-binary and 21 LGBT+ participants (13 did not disclose their sexual orientation). While most were aged 13–18 and organized by year group within the schools, there was also a 20-year-old participant in the focus group recruited via the LGBT+ youth club because the club was open to young people up to age 24.

Young adult interviews lasted 30–60 min and followed a narrative format; participants were asked to describe their circumstances when lockdown was first imposed and were prompted to elaborate on their ensuing experiences. Questions asked included: were you in a relationship before lockdown and/or at any time during lockdown? What was the nature of that relationship (recent, casual, committed, long-distance, etc.) and have there been any changes during or since lockdown? What was lockdown like for you both personally and, if applicable, in your relationships? Those not in relationships were asked about what they wanted in terms of relationships before and during lockdown and the effects of lockdown on how they went about their goals (e.g., regarding ‘dating’). All were asked about how they used technology to maintain contact with actual/desired partners and the types of interactions they had, including intimate/sexual interactions and their feelings about these interactions. Participants were also asked about their reflections on the period and how they think the lockdown has affected them and their relationships both currently and regarding their hopes for the future. Some interviews were held virtually on MS Teams and some in-person in the author’s academic office when rules around socially distanced permitted in-person interviews (in line with university guidance).

Focus groups lasted around 1-h, with adolescents reflecting together about what happened in relationships during lockdown. The methodology enabled participants to ‘set the agenda’, as it were, which bridged gaps in perspectives between them and ourselves and addressed power dynamics and meant we were able to unpack the complexities of the topics with participants through dynamic interaction (as suggested by Morgan & Krueger, 1993). They were held in-person in schools and youth clubs, again in line with rules in place at the time regarding social distancing. Each group comprised four to eight participants who were friends or acquaintances, which has been identified as an appropriate size and composition for discussions about personal or sensitive topics and helps in managing participants and encouraging equal contribution (Punch, 2002). They were mostly mixed gender. Initially, with three school groups, we followed a semi-structured focus group guide, containing questions and prompts for participants to consider perspectives on using technology in their relationships during lockdown organized around a set of broad concepts to be explored (see Knodel, 1993). Participants were asked an initial question about how relationships were affected during lockdown and the ways in which they used technology in their relationships during the period. They were then asked about their perspectives on different forms of and contexts for digital intimacies (spanning consensual to non-consensual acts and positive and negative motivations for and experiences of sexting). They shared their views on why individuals may engage in practices like image sharing, what these practices involve and the risks and opportunities of sharing images. Discussions related both to what happened during lockdown itself and perceptions regarding general patterns of image sharing that occur among and between adolescents.

In these first focus groups, any diversions from the guide were re-routed back to the pre-conceived questions. However, during a focus group at the LGBT+ youth club, participants spoke extensively about a range of experiences with digital intimacies following the initial questions. This free-flowing discussion covered many of the anticipated questions on the guide, albeit in a nonlinear direction that raised some unanticipated further topics and enabled us to create a non-censorious space for discussion (see Curtis et al., 2004). Hence, in subsequent focus groups, we adopted a more flexible approach that allowed more youth-led discussion with some general questioning and prompts to cover the key themes of interest within the guide. The group interview setting was akin to young people’s “natural habitats” insofar as it comprised a “group of mates” (see Frost, 2003). Yet, as facilitators, we were experienced and had undergone training in group interviewing methodology and used questions and prompts to draw out underlying dynamics and norms that characterized participants’ accounts (see Hill, 2006).

All participants’ identities and any identifying features in the data were anonymized, with participants choosing pseudonyms. Confidentiality was upheld with exceptions for safeguarding, although no disclosures were made that required a safeguarding response.

### Analysis

Discussions were audio-recorded and professionally transcribed by a transcription company. Data analysis was undertaken manually by the two authors once all transcripts had been received. A small number of transcripts were subject to initial coding, and we met to discuss and agree emergent codes. Thematic analysis was adopted to identify, analyze, and report patterns (themes) within the data. Following the six stages of thematic analysis (Braun and Clarke, 2006), we familiarized ourselves with the data, generated initial codes, examined differences and commonalities within and across code categories for these transcripts, identified and resolved instances of coder disagreement, and sorted codes into groups under themes, before continuing with independent coding of the remaining transcripts.

Coding was iterative and inductive as we continued to refine the codes with each analyzed transcript. We adopted a constant comparison approach, where segments coded with the same code were compared to ensure they reflected the same concept. We then collaboratively grouped codes to form overarching themes that expressed the latent content of transcripts. We considered coding finalized when no new concepts were identified in the data, suggesting theoretical saturation had been achieved.

While we cross-checked and verified our interpretations of the data, the act of representing the data through common themes involves an element of subjectivity. We did not quantify meanings, perceptions, or experiences because the fact that a theme was not raised in an interview or focus group does not mean it was not of importance, but perhaps that the conversation had taken a different direction. Hence, we cannot attest to prevalence of different perspectives among adolescents or young adults but instead present the findings as illustrative of how participants (co-)constructed and articulated the sociocultural and intra- and interpersonal dimensions to sexting. We note the extent to which particular themes emerged across the sample, stating, for example, whether a theme was raised by most, several or few participants. We do so in respect to this specific sample and to show the full range of perspectives and themes, rather than to make any general prevalence claims about adolescents’ and young adults’ perspectives and experiences.

Themes were interpreted from a critical realist perspective. Ontologically, critical realism understands reality as existing beyond subjective experience but as given meaning at the intersection of “person and society” (Clegg, 2006, p. 317). Wood (2021, p. 636) applies the critical realist concept of “emergence” to technology-related harms to identify the multiplicity of “imbricated strata” within which casual mechanisms reside, whereby, he argues, properties inherent in technology “remain latent until activated by human–technology interactions,” thus requiring an understanding of material and human agency as related rather than conflated.

Lockdown—as an event or condition—did not, from this perspective, cause patterns of digital intimacies but (re)created conditions for action and experience shaped by interplays between these conditions, the properties of technology, and subjective, interpersonal, and sociocultural dimensions of gender, sexuality, and relationships. While these transcend lockdown, lockdown structured how they played out and the reflective and reflexive accounts shared by participants regarding sexting during this period.

## Results

Participants expressed various perspectives on and experiences of synchronous and asynchronous sexting practices. Discussed first are adolescent peer cultures of normalized non-consensual distribution of intimate images and a reluctance to intervene or report in response to incidents of non-consensual distribution. Next, we outline others forms of non-consensual sexting, including unsolicited sexting and unwanted requests for images. We examine adolescents’ continued reluctance to intervene or report and girls’ experiences of turning down requests to sext from boys. We then turn to how young adults narrated their perspectives on sexting during lockdown, with themes pertaining to: sexting as unfulfilling; sexting as impossible or risky; sexting to bridge distance and for self-expression and self-exploration; and willing but unwanted sexting.

### Adolescent Peer Cultures of Normalized Non-Consensual Distribution of Intimate Images

Adolescent participants discussed sexting in terms of the potential for non-consensual further distribution of images by the recipient and the ensuing social and reputational risks to subjects of such images. Kobe (14, M) said images are “leaked…all the time…constantly” at school. Participants typically felt those who “leak” images do so for comedic value, to show off, or seek revenge. Many perceived that it is mostly girls’ images being shared by boys. Some girls said boys “don’t have respect for females…[and they] think it’s funny” (Ruby, 14, F) and are “proud” (Ruby; Emma, 16, F) when showing images of girls to their friends.

Patrik (14, M) perceived a gender double standard whereby sexting is “more embarrassing for girls…Because for a boy it would be like, oh, go on. But for a girl they would be called like s-l-a-g.” Emma said boys’ friends will typically say “well done” to the boy but “if a girl does it, she gets shamed.” Ashley (16, F) bemoaned how “socially, girls are expected to be more…nice and timid, but boys are more arrogant,” while Emma resented how boys “just walk around all cool…I get the feeling that it’s [sexting] not as big a deal for them as it is for us.” Participants believed these gender dynamics underpinned the greater propensity for boys to distribute images of girls and the more damaging consequences to girls.

This social context to adolescent sexting meant participants felt that technical tools embedded into digital platforms designed to prevent privacy violations are partial or ineffective solutions as motivated individuals seek to circumvent them. The Snapchat alert function (which notifies users if their message has been screenshot by the recipient), for example, was deemed by the group below not to fully ameliorate risk because the recipient may find another way to capture the image:

**Table:** 

Whippersnapper (15, F):	And even on Snapchat when you’re alerted when someone has taken a screenshot there must be apps out there where it isn’t notified
Sammy (15, M):	Oh yes, you can take just get a second phone –
Whippersnapper:	Exactly, yes
Sammy:	And take another shot of it

Because of the risk of non-consensual distribution of images and associated stigma, some girls felt any positive experiences of sexting for girls are inevitably “short-term” (Ashley, 16, F). Ashley said that “even if [girls] feel empowered…[sexting] can still be used against them and along the line, they could regret doing that [sharing images].” Scarlett (16, F) framed the risk in terms of precarity arising from the impermanence of trust and a lack of control:

…if you broke up and at the time you trusted them to have the pictures but then when you break up they still have the pictures and you don’t have that same trust with them as you had before but you feel like you can’t just message them and say, can you delete them, cos you can never be certain that they have.

While Ashley described non-consensual distribution as “an attack on dignity,” she said that agreeing to sext generates self-blame among girls: “the next day [following a decision to sext], I would be like, I’m really encouraging this…then you feel guilty.”

### Reluctance Among Adolescents to Intervene or Report Incidents of Non-Consensual Distribution

Adolescent participants described a reluctance to report incidents of non-consensual distribution that they witness or experience. They felt that those responsible for non-consensual distribution are often difficult to identify because images are shared “quickly,” with news traveling through “gossip” (James, 14, M), “people hearing from other people and then those people telling other people” (Jimmy, 14, M), and “most of the time…you won’t see [the image]” (Taylor, 15, gender fluid), which means “you don’t know who originally started sending them around” (Ruby, 14, F). For these participants, the challenges in identifying the person who non-consensually shared the image presented a practical barrier to reporting.

Presumably the incident itself could be reported regardless of whether the original person who shared the image is identified. Yet, concerns about peer inclusion and exclusion inhibited reporting and, moreover, to intervening within the peer group itself. Aye (14, F) said that “if all your mates think [the leaking of the image] is the right thing, then obviously if you say it’s wrong, then all your mates are going to turn against you.” Jimmy (14, M) added that “at the end of the day, you don’t want to not be mates with them over something that has nothing to do with you.” There was seemingly some available vernacular that avoids “a big confrontation” (Aye); for example, saying the behavior is “tight” (Jimmy) or “not cool” (Aye), which these participants felt would be less likely to jeopardize the friendship or one’s social position.

Adolescents were concerned that reporting to adults may make the problem worse. Several participants described adult involvement as awkward and embarrassing. George (15, F) recounted an occasion when police were notified of sexting at her school and described it as “a huge shenanigan.” For victims of non-consensual distribution, Jane (14, F) felt it is commonsensical not to want adults involved because of privacy concerns: “if you’re going to send something to someone, you wouldn’t want anyone to know.”

“Anti-snitch” cultural norms also entrenched a reluctance to report. Aye (14, F) referred to not wanting to be seen as a “snake” or “snitch” because “if someone tells, then everyone will turn on that person.” When asked what is bad about being seen as a snake or snitch, she said that if “everyone is backing each other and there’s one person that tells, well now everyone is in trouble because that one person has snaked.” Jimmy (14, M) added that “all the others are lying to just get out of it. That’s what it’s all about, getting out of trouble…never wanting to get in it.”

### Unwanted and Invasive Image-Sharing and Requests for Images

Adolescent and young adult participants described other forms of non-consensual sexting, including unsolicited image sharing and unwanted/pressured requests for images. Reflecting the gendered landscape of adolescent sexting cultures outlined above, some adolescent girls felt that during lockdown, boys had more “confidence” to send and request images when “behind a screen” (Lola, 16, F) presumably because they did not have to contend with any ramifications in-person. Ashley (16, F) believed such boys felt entitled regarding their perceived “right to do it [pressure girls].” Some girls linked these perspectives to their experiences with boys who send unwanted images. Emma (16, F) said “sometimes they’ll message and say, do you want me to send it, and you’re like, no, and they still do.” Emma believed boys do not necessarily send unsolicited images “as a way of flirting…they just want to do it.”

Resulting from these perceptions was the idea expressed in two groups comprising female adolescents that it is a “red flag” if boys want to sext. They described repeated pestering and indirect coercion, for example boys comparing girls unfavorably to ex-partners. Amelia (16, F) felt this indicates that boys “don’t want you for your character or something, they just want your body…and it feels a bit disrespectful,” with Emma adding that they “don’t care about your thoughts or feelings…[they’re] just treating you lesser than them.”

Despite being critical of boys, girls were reluctant to report being sent or pressured for images. Ammy (17, F) said that if boys “find out you reported it, they have a go at you saying, oh my God, you’re too uptight; why would you do this to my mate…then it would go round school and then I would think, maybe I am too uptight.” Responding to unwanted sexting was also constrained by mitigating risks of further harm. Magda (14, F) described being insulted by boys: “you get violated with different names if you don’t say anything to them.” Amelia (16, F) attributed these reactions to a desire to regain confidence: “it’s an ego or self-confidence thing for [boys]. If you were to say no…then they’ll pin that on you…they’ll insult you or say something just to give themselves more confidence again.”

Girls described turning down unwanted sexting while not antagonizing boys. In one group, there was mention of TikTok videos containing tips on refusing requests in a light-hearted way as well as “fake boyfriend snaps” (Louise, 14, F) which were described as “lifesavers” (Jane, 14, F). Louise explained these are “pictures of like a boy…and it’s like, yes, I can’t, I’m with my boyfriend.” While Emma (16, F) said boys sometimes respond that “it doesn’t matter,” Lola (16, F) said that, typically, “…if you say you’re in a relationship they’re actually more likely to back off…as soon as you introduce another boy to the situation then they’re more likely to be more respectful.”

Adolescent girls also described being sent images from boys and men online who they do not know or cannot identify. A normalization of connecting with new people online among adolescent participants meant Louise (14, F) described accepting requests from strangers because they “want to see what they’ve said” but feeling “sick” when it is an explicit image. Magda (14, F) described requests to sext from strangers as “weird talk” that makes her feel “uncomfortable.” Skye (14, F) said in response she “block[s] them straight away and then it’s done…[but] it plays in your head for a bit, like the amount of times it has happened.” Jane (14, F) described “show[ing] friends’ or, even, claiming to be ‘a 9-year-old, you’ll tell the police [to] get them scared” and “make them apologize” but, ultimately, just “block[s] them.” While unpleasant, responding to these interactions through ignoring or blocking the sender felt possible in contrast with boys they know where there was more emphasis on communicating the refusal in ways that would not be antagonistic.

A young adult woman, Canq (22, F), recounted being sent explicit images after boys at school posted her contact details online. Her experience suggests the images were sent to harass Canq as a targeted form of abuse:

…when I was 15, these boys from another school didn’t like me so they put my Snapchat on a porn website…I just had hundreds of people adding me…the amount of pictures I was getting from these people. It was disgusting…just a picture of their genitals…

Another young adult woman, Carolina (23, F) said she still experiences men sending unwanted images and requests for images online and perceived me to engage in more of these behaviors during lockdown. She described a “kind of repression and not being able to, like, hook up with people…,” suggesting an attribution of these behaviors to men’s sex drives. Canq’s story, however, reflected a more deliberate form of abuse.

### Young Adult Perspectives on and Experiences of Sexting During Lockdown

Young adults held various perspectives on sexting, with some disavowing it while others felt it helped to bridge distance and experience intimacy. There was also some ambivalence apparent. Privacy concerns and gendered relational dynamics of expectation and obligation shaped meanings and experiences among some young adults.

#### Sexting as Unfulfilling

Some young adult participants described themselves as not sexually active. Mary (19, F) and Don (19, M) did not believe in sex before marriage, while Cecilia (20, F) identified as asexual. Sexual intimacy, including sexting, was, therefore, less salient for these participants. Some of those describing themselves as sexually active nevertheless said they were not interested in sexting. Canq (22, F), for instance, expressed desire for in-person sexual intimacy but not sexting: “it doesn’t do anything for me, I don’t want to see it and I would rather have that experience with someone in person and wait…I just don’t get any satisfaction from it over the phone…” Canq suggested that her perspective was somewhat shaped by her experiences of abusive image sharing described above.

Some young adult men were disinterested in sexting, which seemed related to their belief that it does not provide a substitute for in-person intimacy with there being no mention of unwanted or harmful experiences. Alex (22, M), for example, described sexting as unfulfilling, potentially frustrating, because of the lack of physical co-presence:

…I totally turned myself on, but…it was kind of bothering me because you’re [his girlfriend] not here and I’m turned on and we keep on disclosing naughty stuff and you’re not here to help me out…I’m not there to help her out.

Jimmy (20, M) similarly desired intimacy with his girlfriend but described image sharing as indicating a “lack of self-control.” Jimmy’s perspective related to privacy concerns (see below), whereby sexting was deemed to involve a salience of intimacy needs over measured risk management. Jimmy felt the lack of intimacy during lockdown “took a toll…because we…missed each other very much and we couldn’t really do anything about it,” suggesting the desire for intimacy was not the problem but the pursuit of it through risky sexting.

#### Sexting as Risky Due to Privacy Concerns

Some young adults were concerned about privacy. Like the adolescent girls above, Grace (19, F), said that “I am aware that if you send something it then leaves your control…I trust him [her partner] completely, but he can say he’s deleted that picture…and he can keep it, it’s not in my control anymore.” Her insistence that she “completely” trusts her partner was contradicted by her belief that he may say he has deleted the images when he has not, which may relate to her self-described constrained and ambivalent choice to engage in sexting, which is returned to further below.

Others candidly described a lack of trust. Gary (18, M) stated he does not send “naked pictures because I don’t trust her [his partner]…she might definitely want to [share them further]…maybe when we are no more together…” Sending written messages was deemed by some young adults to be “safer” than visual imagery. Amber Valentine (21, F) said she enjoyed sharing messages with her boyfriend “more as a teasing thing” but did “not take the chance and have a recording…because literally anything can happen.” John (23, M) similarly said he and his female partner messaged about what they wanted to do intimately when together in-person. They were not “explicitly like, oh, we will do this when we meet up, but I think this was kind of just like implied [in the communication].”

Somewhat differently, Lily (24, F) described privacy concerns ensuing from her living situation during lockdown. She felt sexting may be “helpful for a lot of people…if you’re physically distanced, being able to communicate that way and have intimacy,” because when “you can’t see each other for so long, like it gets to the point where you need to keep the intimacy.” However, because she and her male partner were living in their “family homes…it [sexting] didn’t really feel that appropriate.”

#### Sexting to Bridge Distance and for Self-Expression and Self-Exploration

*Intimacy and self-expression when apart* Young adults who described engaging in sexting of some kind during lockdown said they wanted to sustain their relationships when physically apart. Francesca (23, F) described sexting with her boyfriend as “a tool that you use to…stay in touch with…the physical side as much as we can…through a screen.” Alison (19, F), likewise, described “camming” (synchronous sexting via videocalls) as “beneficial” when apart from her boyfriend, but as a substitute because she “would rather, like, do it in-person.”

Adolescent participants were not questioned on personal experiences of sexting. While some adolescents raised instances of non-consensual sexting as outlined above, there was little mention of any more positive/willing involvement in sexting. Sarah (14, F), however, described being more “flirtatious” online and said she participated in more “sexual communication over lockdown…” but has “stopped that now that lockdown is over” because she “felt more confident in lockdown” when “it was just messaging, and I didn’t have to see them in real life” and reverted to in-person intimacy after lockdown.

Other adolescents perceived an increase in sexual interactions online during lockdown. Lewis (15, M) attributed this to a desire for intimate connection, which, he felt, meant it has now “slowed down because I think people met in-person…” while Louise (14, F) attributed a perceived increase in young people posting revealing “selfies” (e.g., “in their underwear”) to a desire for compliments and affirmation at a time when people “weren’t getting validation from just going out and seeing people…so, they were getting it from social media instead.”

*Intimate exploration* Ivy (21, F) narrated a process of sexual self-exploration through sexting. She and her boyfriend shared images and had “phone sex” during lockdown. She described herself as previously sexually inexperienced and recounted how “…we kind of struggled initially anyway with our sex life because I [had]…completely no idea what to do or how anything worked…” Sexting was instrumentally valuable for bridging distance for Ivy and her partner while helping them explore what they wanted and enjoyed because they were “forced to communicate.” Ivy found it “really helped in the long run…communicating on video chat really forced us to…look at what we needed from each other…” It has since become “a pretty normal thing for us especially when we’re apart.”

#### Willing But Unwanted or Reluctant Sexting

Some young adult women believed that sexting typically involves girls and women seeking to please boys and men. Lexi (22, F) felt that “guys find sending and receiving pictures a lot more appealing and something that is actually sexual for them whereas for girls it’s more of a hassle and just something that they do to humor the guy.”

Lucy (20, F) believed that her boyfriend enjoyed sexting during lockdown more than she did. She described him as more visually oriented than her and said she did it to please him: “I’d send photos every now and then…he didn’t like force me to obviously, but it was mostly for his sake rather than any enjoyment I got out of it.” While she did not define it as non-consensual, she narrated an obligation to please him regardless of her personal desire. She said that now they can see each other in-person, she no longer experiences this feeling of obligation, again underscoring the primacy of in-person intimacy.

In contrast, Grace (19, F) described having set an expectation for sexting with her boyfriend during lockdown, which has persisted despite now being able to see him in-person. She felt “uncomfortable with [sexting]” during lockdown but obligated to “help” him: “I felt sorry for him…he needed something like that…I probably did that more for him than myself.” Grace said that sexting during lockdown has had “a knock-on effect…now that he’s experienced that…He likes the pictures…so that’s kind of continued.” Like Lucy, Grace insisted her partner was not “intentionally pressurizing” and would not want her “to feel pressured.” She said it was her “choice” to please him, but that not doing so risked reducing the longevity or faithfulness of the relationship because “if I sent this [an image] to him then…he’ll be happy and he’ll stay in the relationship a bit longer…He won’t have a wandering eye because [he] won’t need to have one.”

There was less evidence of the young adult women expecting their male partners to accede to their desires and no talk of these dynamics unfolding within same-sex or gender fluid relationships. Alyssa (24, F), for example, said she would like to sext with her boyfriend, but they do not because he does not want to. Yet, Mikey (20, M) described experiencing pressure to please his girlfriend despite his privacy concerns: “…sometimes when we are far apart, she says, Babe, come on, okay just snap and send for me, and if I’m snapping it, I sometimes, said okay. I did it twice, but I didn’t show my face…”

Two young adult women—Ellie (22, F) and Lisa (22, F)—, who participated as friends in a paired interview, spoke about Ellie sending unsolicited images to her boyfriend. Ellie said he sometimes expressed annoyance, for example if he was at work, but Ellie and Lisa described his annoyance as funny rather than a violation of his consent. They also said it was okay because Ellie and her boyfriend “trust” one another, meaning they deemed him unharmed by these seemingly unwanted intrusions.

## Discussion

Perspectives and experiences of sexting shared by adolescents and young adults in the focus groups and interviews were shaped by both the respective methodological stances and age ranges of the participants. Notably, adolescent participants co-constructed social and cultural norms and meanings about sexting in ways that reflected what has been found in previous research; adolescent girls perceived and described a gender inequitable landscape of risk and reward in peer sexting cultures that, they believed, operate to the detriment of girls while enabling boys to accrue value through engaging in sexting (e.g., Ringrose et al., 2021; Setty et al., 2022). The young adults, meanwhile, articulated (inter)personal perspectives on and experiences of sexting that both reproduced and transcended these sociocultural constraints.

Several adolescent girls in the sample felt that girls should be cautious of sexting and, perhaps, that boy’s interest in sexting denotes a “red flag,” because it indicates objectification rather than genuine interest and previous studies suggest that boys also believe that girls will be disadvantaged by sexting so counsel them against it (Setty, 2020). From this perspective, sexting may, at best, only be experienced positively by girls in the “short-term” amid self-responsibilization for managing the risks of non-consensual and abusive sexting in a landscape of uncertainty and precarious trust (see Doyle et al., 2021). There is little space within this gendered landscape of risk for girls to identify or articulate any benefits from sexting (Setty et al., 2022).

The focus group environment inhibited the articulation of different perspectives on sexting, with shame, risk, and stigma dominating the discussions. Focus groups typically involve co-construction of social meanings based on perceptions of the phenomenon under discussion rather than the sharing of personal experiences (Crofts et al., 2015). The gendered and heteronormative adolescent sexting culture that emerged in this study both reified a gender inequitable heterosexual dynamic whereby girls carry risk and boys are rewarded, while rendering invisible the perspective of LGBT+ young people, boys who experience harm, and girls who have positive experiences. The heteronormativity may relate to adolescent perceptions that same-sex couples are inherently more considerate of each other and have shared interests, and so are more likely to engage in mutually rewarding intimate interactions and less likely to ‘betray’ each other through non-consensual acts (Setty, 2020), a perception that is unlikely to accurately depict all same-sex sexting, especially given evidence regarding the increased risks that LGBT+ young people face in sexting culture (Albury & Byron, 2016; Needham, 2021).

There was extensive discussion among adolescents about a reluctance to intervene and/or report incidents of abusive sexting to adults. This reluctance stemmed from peer cultural norms discouraging intervention and reporting, whereby the desire to maintain/achieve social inclusion and avoid being labeled a “snitch” superseded any willingness—at least in terms of what they felt able to say in the group environment—to report or intervene, as also found by Harder (2021). Adults were deemed to make matters worse (also see Dodge & Lockhart, 2022; Jørgensen et al., 2019; Ringrose et al., 2021; Quayle & Cooper, 2015; York et al., 2021) and witnesses seemed at most willing to tell peers they were being “uncool.” There were, furthermore, concerns among girls about antagonizing boys who engage in non-consensual sexting. There was some tempering of any resistance, with girls describing carefully, gently, and sometimes innovatively resisting boys’ requests to sext, as found by Thorburn et al. (2021). Whether non-consensual sexting is experienced or witnessed, therefore, the response was constrained through concerns that any response may cause them, or their peers, further harm.

While showing some innovation when responding to unwanted images and requests for images, some responses described by the girls raise concerns. For example, while some felt they benefit from the protection of a ‘faked’ heterosexual relationship when refusing boys’ requests, this tactic reflects and reinforces heteronormative gender inequalities that delimit girls’ rights to make autonomous choices for themselves and so may not represent a full or just solution. Moreover, digital tools—like the Snapchat “screenshot” alert or the ability to block/ignore unwanted interactions—do not address the wider contexts of these interactions. Digital tools and resilience do not, therefore, protect or provide a solution to the gendered harms of non-consensual sexting (Meehan, 2022).

The scope for more nuanced sexting experiences became evident in the accounts of some of the young adults who, by virtue of the interview methodology, narrated more personal and interpersonal orientations to sexting. Those disinterested in sexting often referred to feelings of desire (for intimacy), which they felt could not be met—or may even be frustrated by—sexting. Sexting may, therefore, generate anticipation for sexual activity (Johnstonbaugh, 2022) but an inability to physically satisfy arousal in-person with partners during lockdown was the problem for these participants. There were concerns about privacy, but these were typically articulated through relational dynamics of trust rather than gendered and heteronormative stigmas, as was the case for the adolescents. Yet, such stigmas may implicitly underpin privacy concerns, suggesting it is at the intersection of sociocultural contexts, interpersonal relationships, and subjectivity that privacy concerns emerge. Lily’s concerns about a lack of privacy when living with family, meanwhile, was consistent with findings regarding lower rates of sexting among young people confined to the family home (Romero-Rodríguez et al., 2022).

Several young adults described volitional sexting from which they took varying levels of pleasure, as has been found in other studies (e.g., Johnstonbaugh, 2022). Sexting operated instrumentally to bridge physical distance and offer mediated intimacy in absence of opportunities for in-person intimacy, consistent with findings from Bianchi et al. (2021). Privacy concerns co-occurred alongside a desire for intimate connection (Thomas et al., 2022) and there was a normalization of the need/desire for intimacy (see Bonilla et al., 2021). Ivy narrated a process of (inter)personal intimate and sexual exploration through sexting during lockdown which could be deemed instrumental because it was described as aiding self-knowledge and interpersonal connection in ways that support a “physical” sex life. Sexting also, however, seemed to hold meaning in and of itself as a form of intimacy for Ivy. Moreover, like those desiring intimacy in the absence of physical contact, sharing sexual content online was deemed to potentially have helped individuals ‘feel seen’ by others. One of the adolescents perceived this to be a motivation for posting intimate selfies online that aligns with evidence regarding the intra- and interpersonal dynamics of identify exploration and development during adolescence (Collins et al., 2009).

Some young adults described experiences of sexting whereby consent seemed not to be fully present. It was mainly young adult women narrating feelings of obligation to engage in sexting that was unwanted or about which they felt ambivalence, as found in other studies (Amundsen, 2022; Bonilla et al., 2021; Johnstonbaugh, 2022; Laird et al., 2021). The relational context meant they did not describe the pressure as direct but instead as arising from a fear of losing, or at the very least disappointing, their partner, which could represent indirect coercion (see Agnew, 2021). The idea that sexting is a substitute for in-person intimacy meant these feelings were constrained to the lockdown period for Lucy, but for Grace seemed to have created an ongoing expectation that was difficult to address.

While agreement to unwanted sexting seemed to remain a gendered phenomenon among the young adults, the findings from the interviews overall suggest that when articulating (inter)personal subjectivity and experience beyond a group environment, gender double standards and inequalities become somewhat less powerful and may be transcended personally and/or interpersonally. Some young women described volitional and wanted sexting and several young men claimed disinterest in sexting, contrary to assumptions about gender and desire. There was also an account of a young man—Mikey—engaging in unwanted sexting, while Ellie and Lisa downplayed the harm of Ellie’s unsolicited image sharing to her boyfriend, underscoring the importance of recognizing, rather than trivializing, the ways that sexting may be detrimental to men (see Agnew, 2021; Hunehäll Berndtsson & Odenbring, 2021; Ravn et al., 2021; Setty, 2020).

Disentangling group-level norms and meanings as constructed in focus groups from individual experience (Crofts et al., 2015) helps in showing how the former may shape, but not determine, the latter. Furthermore, young adults may have developed (and continue to develop) a (inter)personal positionality on sexting beyond the demands of adolescent peer culture. It is during adolescence that social policing, and, in turn, the dynamics of stigma, shame and reward are particularly heightened and, therefore, may shape what is said within the focus group environment. Social norms and meanings seemed to affect how adolescents related to sexting, with it being difficult to tease out exactly what constitutes free choice for adolescents, especially for girls but also boys (see Setty et al., 2022). Previous research has found that both boys and girls described a pressure to adhere to sociocultural norms and expectations through their sexting choices, which sometimes creates conflicting demands, for example regarding girls needing to be responsive to male sexual interest while preserving their reputations (Ringrose et al., 2012; Setty, 2019; Thomas, 2018). While young adults may still be grappling with these norms and meanings, the sociosexual developmental process that unfolds through adolescence and into young adulthood may involve increased opportunities for (inter)personal sexual intimacy and self-expression. These processes may work to both entrench and rework gender and heteronormative patterns of meaning and experience.

As a result, disentangling—but recognizing the intersections between—subjectivity, interpersonal relations, and sociocultural norms and meanings, including through complementary methodologies, enables an understanding of the conditions in which choices are made and risks, opportunities, harms, and rewards, arise, and, in turn, how to overcome and make space for ethical digital sexual cultures and digitally mediated sexual interactions, both for adolescents and young adults.

### Limitations

Participants in this study were not representative of all adolescents and young adults in England. Through not quantifying the findings, we cannot attest to the prevalence of each perspective or experience. Instead, we offer the different perspectives and experiences as examples of the ways in which participants (co-)constructed meaning about sexting and what these (co-)constructions suggest about the conditions of consensual and non-consensual sexting. Further targeted research should explore how widespread and normalized the different perspectives and experiences are, including pertaining to issues not raised or represented by participants, including experiences of same-sex sexting contexts.

There were methodological limitations. Interviews generated individual-level accounts, which we could not situate in terms of the contexts and significant others to which participants referred. Focus groups involved participants co-constructing meaning, but wider contexts to which they referred were not accessible, nor were perspectives or experiences that participants did not wish to share in the group setting. Finally, the data pertains to participants’ subjective understandings and perspectives, which, while important, cannot be used to make inferences about any verifiable reality.
